# Rogue Sperm Indicate Sexually Antagonistic Coevolution in Nematodes

**DOI:** 10.1371/journal.pbio.1001916

**Published:** 2014-07-29

**Authors:** Ronald E. Ellis, Lukas Schärer

**Affiliations:** 1 Department of Molecular Biology, Rowan University SOM, Stratford, New Jersey, United States of America; 2 Evolutionary Biology, Zoological Institute, University of Basel, Basel, Switzerland

## Abstract

Intense reproductive competition often continues long after animals finish mating. In many species, sperm from one male compete with those from others to find and fertilize oocytes. Since this competition occurs inside the female reproductive tract, she often influences the outcome through physical or chemical factors, leading to cryptic female choice. Finally, traits that help males compete with each other are sometimes harmful to females, and female countermeasures may thwart the interests of males, which can lead to an arms race between the sexes known as sexually antagonistic coevolution. New studies from *Caenorhabditis* nematodes suggest that males compete with each other by producing sperm that migrate aggressively and that these sperm may be more likely to win access to oocytes. However, one byproduct of this competition appears to be an increased probability that these sperm will go astray, invading the ovary, prematurely activating oocytes, and sometimes crossing basement membranes and leaving the gonad altogether. These harmful effects are sometimes observed in crosses between animals of the same species but are most easily detected in interspecies crosses, leading to dramatically lowered fitness, presumably because the competitiveness of the sperm and the associated female countermeasures are not precisely matched. This mismatch is most obvious in crosses involving individuals from androdioecious species (which have both hermaphrodites and males), as predicted by the lower levels of sperm competition these species experience. These results suggest a striking example of sexually antagonistic coevolution and dramatically expand the value of nematodes as a laboratory system for studying postcopulatory interactions.

## Abstract 2

Reproductive competition often continues long after copulation ends. This article reviews the field and introduces a new paper showing that nematode sperm can directly harm the female germ line.

*On the Origin of Species* focused almost exclusively on the role of natural selection in evolution [1], but Darwin realized that animals also compete for mates and described the process of sexual selection at length in a later book [2]. The simplest examples involve combat like that between male elephant seals fighting for access to females. However, sexual selection also includes many other types of interactions. For example, some male birds have elaborate plumage because females favor this trait when choosing mates (reviewed in [3]). In their simplest form, these interactions can be thought of as parts of a triangle—competition between two males forming the base and the interactions between each of the males and the female forming the two legs.

## Many Types of Sexual Selection Occur after Copulation

It took almost a century for scientists to realize that sexual selection often continued after copulation was finished [4]. If several males mate with the same female (which appears to be the rule rather than the exception), their sperm will often compete within her reproductive tract for access to her oocytes. During mating, males therefore try to enhance the success of their own sperm by actively displacing those from previous males, by chemically preventing females from further mating, or by making sperm that are competitively superior in number or phenotype (reviewed in [5],[6]). Because these interactions are played out inside the female, she will often influence and sometimes control the postcopulatory actions of both her mates and their sperm, giving her opportunities to favor the use of one male's sperm over another. This bias is often termed “cryptic female choice” and represents the postcopulatory equivalent of more familiar types of female choice (reviewed in [7],[8]).

## Postcopulatory Sexual Selection Can Lead to Conflicts between Males and Females

Since male-male competition often selects for ejaculates that are more abundant or more persistent than desirable from the female's perspective, females often evolve countermeasures that control what males (or their ejaculates) can do to them, which in turn favors male traits that can overcome these female defenses. This sexual conflict between mates following insemination results in ongoing sexually antagonistic coevolution. Striking examples include manipulative seminal fluids in fruit flies [8], sperm with bristles in some free-living flatworms [9], and traumatic insemination that bypasses the normal route of fertilization in a whole range of organisms [10]. Similar phenomena also occur during sperm-egg interactions in free-spawning marine organisms and in many cases have led to striking patterns in the evolution of the morphologies and molecules that mediate these interactions [8],[11].

## Evolutionary Biology of *Caenorhabditis*

**Figure 1:**
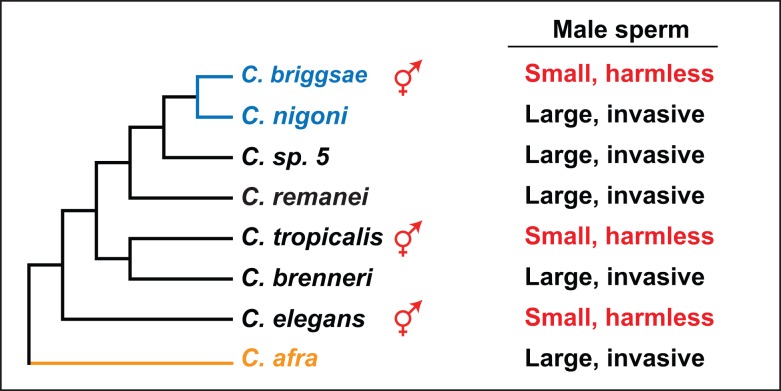
Hermaphrodites have evolved in three independent lineages in *Caenorhabditis.* Only species with sequenced genomes are shown. Androdioecious species (comprised of males and hermaphrodites) are marked with a red symbol, and the others are dioecious (comprised of males and females). The two species in blue are able to interbreed and produce fertile offspring, and the outgroup for the elegans group is orange. Modified from Kiontke et al. [14] and Félix et al. [38]. See main text for details on the types of male sperm.

Although the nematode *Caenorhabditis elegans* originally became famous for developmental genetics [12], its genus is now an important model for evolutionary research (reviewed in [13]). Because field studies are still difficult [14], most of that work is done by comparing gene functions in the laboratory, either between different wild isolates or across species. These studies have become easier with the availability of many newly discovered species, nine genome-sequencing projects (Figure 1), and the widespread availability of RNA interference [15] and gene-editing techniques [16]. However, the most valuable feature is hermaphroditic genetics.

## Self-Fertile Hermaphrodites

*C. elegans* and two related species produce self-fertile, sequential hermaphrodites. Such *XX* individuals are female in most respects but can make their own sperm early in life and use them later for self-fertilization. The same species also make *XO* males, which mate with the hermaphrodites in a sexual system called androdioecy. However, males are infrequent in the wild, and outcrossing is less common than selfing in these androdioecious species [17],[18]. Since most other *Caenorhabditis* species are dioecious (comprised of males and females), self-fertility must have arisen on three independent occasions (Figure 1) [14],[19],[20]. The existence of hermaphrodites has been of great utility because it (1) simplifies genetic research in *C. elegans* and *C. briggsae*, (2) provides an important trait to follow in evolutionary studies, and (3) allows direct tests of many theories about sexual reproduction and behavior.

## Sperm Competition in Nematodes

**Figure 2:**
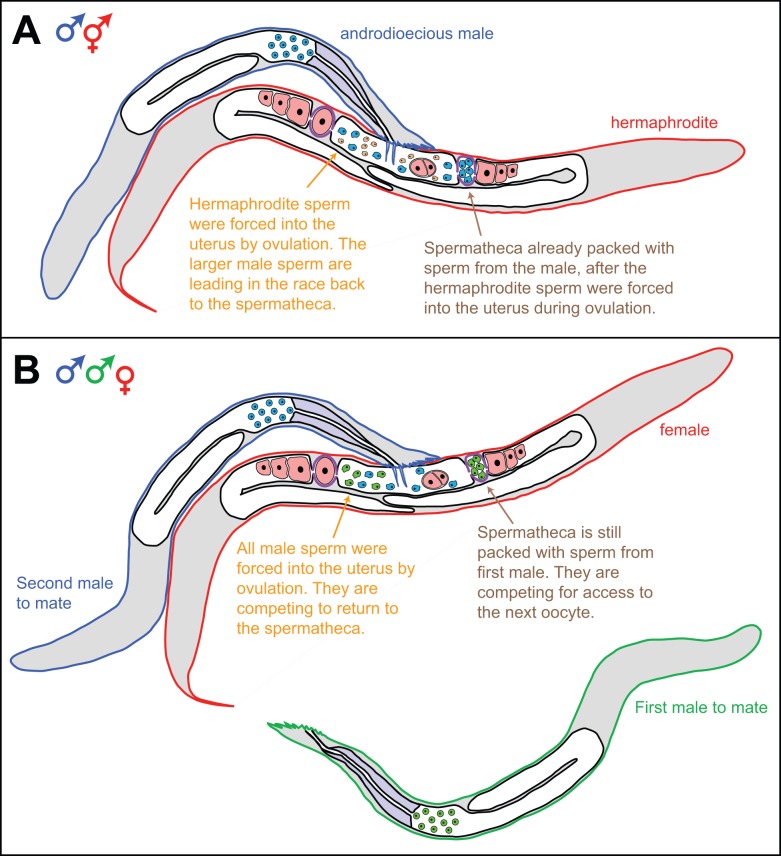
In *Caenorhabditis* nematodes, sperm compete for position in the spermathecae. (A) Diagram of a hermaphrodite (red) mating with a male (blue). The hermaphrodite gonad is bilaterally symmetric with a central uterus. The male is ejaculating larger sperm (blue) into the uterus, and they outcompete the smaller hermaphrodite sperm (pink) in the race to repopulate each spermatheca (purple) after sperm are dislodged during ovulation. (B) Diagram of a female (red) mating sequentially with two males (one green and the other blue). The female's gonad resembles that of the hermaphrodite in (A). The spicules from the blue male have penetrated the vulva, and he is ejaculating (blue) sperm into the uterus. These sperm will compete with those from the first male (green) for positions in the two spermathecae (purple), where they wait for the chance to fertilize oocytes. Although the sperm from the first male have already taken the best positions (shown in the right spermatheca), they will be displaced into the uterus each time an oocyte is ovulated (shown in the left spermatheca) and must compete with those from the second male to reestablish their positions. Although displacement has been directly observed, additional factors that remain unknown might help influence competition among these sperm.

One of the best examples of such a test involves sperm competition. When *C. elegans* males mate with hermaphrodites, their sperm take precedence, and the hermaphrodite sperm are largely excluded from the two spermathecae (Figure 2A); this pattern of sperm precedence is due to the superiority of the male sperm themselves, rather than timing factors or seminal fluid [21]. The advantage could be due to size, since male sperm are larger than hermaphrodite sperm in all three androdioecious species of *Caenorhabditis* [22],[23]. Supporting this idea, direct competition between wild isolates with different sperm sizes showed that males with larger sperm outcompete those with smaller sperm [24], and experimental evolution in conditions that favored multiple matings led to a rapid increase in the size of male sperm within a few dozen generations [25]. Thus, classical studies with *C. elegans* provide strong evidence for sperm competition between males (Figure 2B) and for the superiority of male sperm over hermaphrodite sperm. Because *C. elegans* males appear to be rare in the wild, multiple matings by hermaphrodites should be uncommon, and sperm competition is probably weaker than in dioecious species.

**Figure 3:**
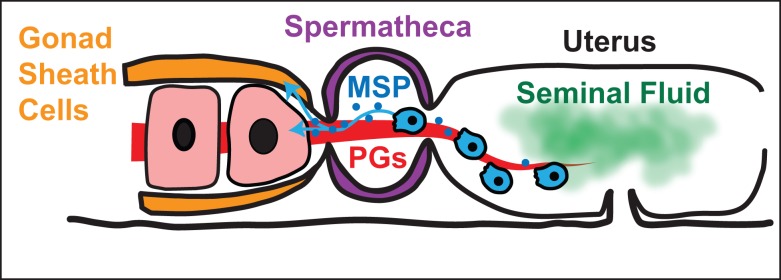
Male and female cues involved in gamete behavior. The male ejaculates sperm (blue) and seminal fluid (green) into the uterus. The seminal fluid contains signals that activate the sperm so that they extend pseudopods and are able to crawl. The seminal fluid is probably complex and might contain additional signals. In addition, active sperm release small membrane-bound packets (blue circles) that contain major sperm protein (MSP), which stimulates oocytes to mature. MSP also causes the gonad sheath cells (orange) to contract and force oocytes through the distal spermathecal valve into the spermatheca (purple). Finally, the oocytes release a complex mixture of prostaglandins (PGs) that guide the sperm. Each of these signals has the potential to mediate sperm competition and/or cryptic female choice.

Studies from *C. elegans* revealed additional factors that might be involved in postcopulatory sexual selection, although they have yet to be studied in an evolutionary context (Figure 3). First, oocytes are involved in attracting sperm towards the spermathecae, and their absence causes sperm to wander aimlessly [26]. This attraction depends on a complex mixture of prostaglandins that is secreted by the oocytes [27]. It would be fascinating to know if the spectrum of prostaglandins has changed during *Caenorhabditis* evolution and if any of these molecules play a role in cryptic female choice. Second, sperm release small membrane-bound packets that contain major sperm protein, which stimulates oocytes to mature and the somatic gonad to contract during ovulation [28],[29]. It is possible that these packets contain additional signals too. Thus, male and female components engage in complex physical and molecular interactions after mating, and these interactions might be under strong selective pressure.

## Interspecies Crosses and Postcopulatory Sexual Selection

Interspecies crosses provide a powerful technique for studying these reproductive interactions [30] and are used with great success in a new study by Ting et al. [31]. Animals from a wide variety of *Caenorhabditis* species can easily be induced to mate with each other in the laboratory; some crosses do not result in fertilization, others lead to inviable offspring, and a few produce hybrids [32]–[34]. Particular attention has been focused on the androdioecious species *C. briggsae* and the dioecious species *C. nigoni*, which are so closely related that they can produce fertile offspring [35].

Now, Ting et al. report a remarkable discovery—in many cases when males from one *Caenorhabditis* species mate with hermaphrodites or females from another, they significantly decrease their fertility [31]. This effect is caused by two distinct interactions. First, these male sperm often physically displace other sperm from the two spermathecae, as expected from previous observations of crosses between *C. elegans* males and hermaphrodites. Second, sperm sometimes invade the ovary, where they induce premature maturation of young oocytes and disrupt the development of new gametes. Occasionally, such ectopic sperm even cross a basement membrane and escape from the gonad altogether. The fact that *C. brenneri* and *C. nigoni* males are equally effective at displacing *C. tropicalis* sperm but *C. brenneri* males more severely affect fertility suggests that both types of interactions matter.

Some of the species in these experiments were androdioecious, whereas others were dioecious. Since sperm competition should be less intense in androdioecious species, observing how they respond in these crosses should provide a strong test for whether postcopulatory selection is involved. Indeed, the authors found that hermaphrodites were much more susceptible to harmful sperm than females. Furthermore, males from androdioecious species made the least harmful sperm detected in any of their crosses. Thus, competition between male sperm is probably at the root of the phenomena they describe.

**Figure 4:**
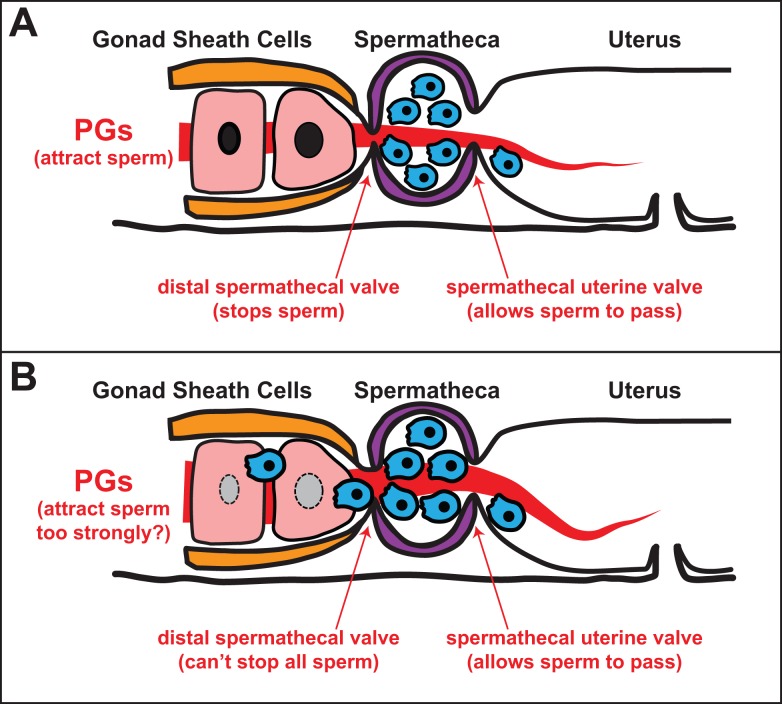
Sperm vigor and female defenses undergo sexually antagonistic coevolution. (A) In a benign interaction between the sexes, male sperm compete for position in the spermatheca, and a combination of female signals and control of the distal spermathecal valve prevent them from entering the ovary. (B) Because of sperm competition, some males develop more competitive sperm that cannot be excluded from the ovary and thus become invasive; resulting fitness costs will favor additional female countermeasures through sexually antagonistic coevolution.

Finally and perhaps most intriguingly, the authors found that male sperm sometimes go astray even in crosses between males and females of the same species. Thus, the interspecies crosses may simply provide a more sensitive way to measure interactions that are going on within individual species in the wild. A simple model that can explain their results is that dioecious males are under intense selection to produce highly migratory sperm, which will have the best chance to find good positions in the spermathecae for fertilizing oocytes (Figure 4A). However, the aggressiveness of these sperm means that females need to develop appropriate countermeasures, such as changes in their chemical signals or the physical strength of the distal spermathecal valve, to keep the sperm contained and prevent them from entering regions of the female gonad where they could cause harm. If the competitiveness of a male's sperm is not in sync with the countermeasures of his mate, some overzealous sperm could go rogue, causing a significant loss in fitness (Figure 4B). Thus, selection on males could favor highly migratory sperm that outcompete those from other males, even if they occasionally lower female fertility. In addition, selection in females should favor protective countermeasures that restore normal fertility but may decrease male fitness. This sexually antagonistic coevolution is expected to cause rapid changes in both sexes, which are revealed when animals from different species are used in experimental crosses.

## Outlook

These studies show not only that nematodes undergo sperm competition but also that additional postcopulatory interactions are significant and appear to include sexually antagonistic coevolution. Given the broad range of interactions between sperm, oocytes, and chemical and physical cues inside the female reproductive tract (Figure 3) (reviewed in [36]), nematodes in the genus *Caenorhabditis* could provide valuable models for investigating postcopulatory sexual selection and sexual conflicts in the laboratory.

New isolates of different *Caenorhabditis* species are being established at a considerable rate (e.g., [14]), which should also permit study of postcopulatory mechanisms in a variety of wild populations. The detection of rogue sperm in some intraspecies crosses might be explained not only by the greater aggressiveness of sperm from dioecious species but also by the high levels of genetic diversity in these species [37], which should make it easier to detect mismatches between male persistence and female countermeasures. Thus, it would be fascinating to analyze the role that genetic variability in dioecious species plays in the range of sperm phenotypes and interactions and to see if the low levels of diversity in androdioecious species limit this range in the wild. If genetic diversity is important, it might even be possible to find specific strains of androdioecious species that produce ectopic sperm in crosses. Finally, we might be able to learn if some sperm traits and female countermeasures are restricted to isolated populations, which genes underlie these traits, and how fast these types of coevolutionary changes are occurring, at morphological, biochemical, and molecular levels. The combination of experimental and field approaches available to address these questions promises an exciting future.
